# Family perspectives in lynch syndrome becoming a family at risk, patterns of communication and influence on relations

**DOI:** 10.1186/1897-4287-10-6

**Published:** 2012-05-25

**Authors:** Katarina Bartuma, Mef Nilbert, Christina Carlsson

**Affiliations:** 1 Department of Oncology, Institute of Clinical Sciences, Skane University Hospital, Lund University, Lund, Sweden; 2 Clinical Research Centre, Hvidovre University Hospital, Copenhagen University, Hvidovre, Denmark; 3 Department of Oncology, Barngatan. 2b, Lund University, 221 85, Lund, Sweden

**Keywords:** Family, Relations, Communication, Lynch syndrome, Qualitative analysis

## Abstract

**Background:**

A growing number of individuals are diagnosed with hereditary cancer. Though increased levels of anxiety and depression have been demonstrated around the time of genetic counselling, most individuals handle life at increased risk well. Data have, however, been collected on individual basis, which led us to focus on family perspectives of hereditary cancer.

**Methods:**

Lynch syndrome represents a major type of hereditary colorectal and gynaecological cancer. We preformed open-ended interviews with 27 informants from 9 Lynch syndrome families. Inductive content analysis revealed three major themes: *transition to a risk family, patterns of communication* and *influence on family relations and individual roles*.

**Results:**

Family members described how learning about Lynch syndrome shifted focus from daily issues to concerns about cancer. Changes in communication related to difficulties in talking to children about heredity and informing new family members and distant relatives about an increased risk of cancer. Influence on relations was exemplified by family members taking on different roles, e.g. females often being responsible for coordinating information about heredity and providing support. Families in which members had experienced cancer at young age typically informed children soon after learning about heredity and at young age, whereas families with experience of cancer at higher age postponed information and thereby also genetic counselling.

**Conclusions:**

Three major family perspectives are described in Lynch syndrome families; becoming a risk family, patterns of communication and influence on family relations. Since these issues are central, our findings suggests that such family perspectives should be considered during genetic counselling in order to contribute to information spread, help family members cope with the increased risk, and motivate family members at risk to undergo surveillance.

## Background

Lynch syndrome is one of the most common causes of hereditary cancer [1] with particularly high risks for colorectal cancer (50–80%) and endometrial cancer (40–60%) [2,3]. The syndrome is characterized by early age (mean 45 years) at onset, but age at onset varies, which implies that individuals in the same family may be affected at young age as well as at higher age. Moreover, 1/3 develops more than one cancer, whereas at least 1 on 5 remain healthy despite the high risk of cancer. Identification of Lynch syndrome families is crucial since surveillance programs efficiently reduce morbidity and mortality from cancer [4-6]. Studies of the psychological impact of hereditary cancer demonstrate temporarily increased anxiety after genetic counselling with most patients thereafter returning to base-line. Some 10% of the patients, however, find knowledge about hereditary cancer difficult to handle and report an impact on e.g. sense of coherence and self-concept [7].

Though information about a disease predisposing mutation has bearing not only for the individual, but also for family members, studies on the psychological effects of hereditary cancer have considered the individual’s views, which led us to investigate family perspectives of Lynch syndrome. Whether affected or not, family members will have to deal with information about heredity, worry for cancer development and concern for children. Information about an increased risk of cancer is generally communicated by family members, which makes information sharing vulnerable to personal interpretations and family relations [8,9]. A number of factors facilitate information spread, e.g. open communication, positive attitudes, well-functioning relations, and support from the partner [8,10]. In contrast, suboptimal communication, poor family relations and decisions to restrict disclosure of information may hamper information spread [9,11]. Family structure and function may not only influence how family members receive information, but also how knowledge about an increased risk is handled, decisions related to genetic testing and participation in surveillance [8,10,12]. With the aim to explore family perspectives, we interviewed 27 members of Lynch syndrome families with focus on how family members perceived that knowledge about hereditary cancer has affected the family.

## Methods

### Informant recruitment

Families that had undergone genetic counselling and testing with identification of disease- predisposing Lynch syndrome mutation were eligible for the study. An invitation letter, containing a confidentiality agreement with information about the voluntary nature of the study, was mailed to the mutation carriers who were asked to forward the invitation to partners and young adults (above 16 years) in the family. Mutation carriers from 12 different families responded to the invitation and 27 members from 9 families were included (Table 1). The mean age among the informants was 45 years (range 18–67 years). 14 individuals at risk of carrying a disease predisposing mutation had undergone genetic counselling mean 6 (2–9) years prior to the interviews. The individuals differed in marital status (married, cohabiting, single, divorced, widowed), parental experience (having children or not, children living at home, grandchildren), mutation status (mutation carrier, non-carrier, not tested) and personal history of cancer. In 5 families (1, 5, 6, 8 and 9) children or young adults had experienced cancer in their parents (before age 24).

**Table 1:** Participants’ characteristics

Characteristic	Number
Families	9
Households	17
Mutation carriers	11
Tested non-carriers	3
Young adults (not yet tested)	3
Spouses	10
Female	13
Married/cohabiting	24
Single	1
Divorced	6
Widowed	2
Parents with children	23
Informants with children living at home	18
Informants with grandchildren	6
Personal history of cancer	7

### Data collection and analysis

Open-ended interviews were conducted by KB and CC, neither of whom had been involved in genetic counselling. The majority of the informants were interviewed in a non-hospital setting and the interviews lasted 15–60 minutes. The initial question was “Can you tell me how heredity has affected your family, as you see it?” and was intended to encourage the informants to tell their personal story without the involvement of the interviewer. The interviews were audio-taped and then transcribed verbatim by a secretary and analyzed by inductive content analysis [13-15]. The text was sorted with the NVivo® software (NVivo qualitative data analysis software; QSR International Pty Ltd. Version 8, 2008). The interviewers (KB and CC) repeatedly read and re-read the transcribed texts and marked selected texts corresponding to the aim, and thereafter grouped these texts together based on their content under preliminary codes. The text under the codes was re-read and subthemes were assigned based on the content. At this point subthemes merged with others or new subthemes emerged. The themes and here under subthemes were finalized by all authors. Informants’ quotes (identified as family/informant, mutation status and age) are used to illustrate the content. Approval of the study was granted by the Lund University Ethics Committee (346/2007).

## Results

Three major themes were identified: transition to a risk family, patterns of communication and influence on family relations and individual roles.

### Transition to a risk family

Even though members of several families had suspected heredity before the identification of a mutation, verification hereof was described to shift the focus from daily life issues to worries about cancer. The subthemes identified were related to how *experience influences risk perception and motivates genetic testing* and how *an increased risk of cancer in children is handled*.

#### Experience influences risk perception and motivates genetic testing

Risk perception was described to be influenced by experience of cancer in the family and shaped the family members expectations. A young adult described expecting to be affected by cancer at a high age, since her grandparents and other relatives had been older at the time of diagnosis.

"“My grandfather died of cancer as well. So I don’t know, I’ve always thought that when you get old, you get cancer and then you die” (3/27, not tested, 20 years)"

A father described how his daughter recognized the same symptoms of colon cancer as he had, which indeed led her to diagnosis:

"“/…/then we had the problem of our daughter starting to have stomach aches too/…/she was 24 years old/…/she could see the same symptoms that I had had” (9/24, mutation carrier, 62 years)"

A major motivator for genetic testing was concern for children. Some mutation carriers reported that the result of genetic testing could affect family planning. The decision to undergo genetic testing was also influenced by family experiences. Individuals in families with many affected relatives or where parents or children were affected at a younger age chose to undergo genetic testing at a young age, typically shortly after the identification of a disease predisposing mutation. In contrast, young individuals in families with few cases of cancer or families in which cancer had developed at an older age reported postponing genetic testing and surveillance:

"“You hear sometimes when my father goes to these kinds of examinations/…/yes that’s right…you have to check out whether you have this gene too!/…/then you forget about it just as easily again. Because it’s been this way since you were little so yeah, yeah it’s nothing for me, I’ll check it when I get older/…/” (3/26, not tested, 22 years)"

#### Handling an increased risk of cancer in children

All parents expressed concern for their children, including worries about cancer being diagnosed at a young age, participation in surveillance programs, frustration over young adults choosing not to undergo genetic testing and difficulties related to respecting independent decision:

"“I guess I was mostly concerned about passing it on to [daughter]. And if you couldn’t … why I couldn’t test my child for the mutation/…/That I would have to wait, because she might get cancer before she turns 18/…/It’s pretty easy to take a blood sample.” (17/6, non-carrier, 37 years)"

Whereas some parents were determined that children should be tested at age 18, others acknowledged their autonomy. The spouse of a deceased mutation carrier described how she was initially convinced that her children should be tested at age 18, but after genetic counselling grasped the complexity of the issue and realized their need for independent decisions in order to be able to handle the result.

### Patterns of communication

All family members discussed how the increased risk of cancer was communicated in the family, reported *strategies for informing family members* and described *communication as open or restricted.*

#### Strategies for informing family members

Family members described difficulties in grasping the complexity of the issue and correctly forward information obtained during genetic counselling. Misconceptions were reported and can be exemplified by a father who was keen to test his son, although he was not a carrier, and a mother who referred to the many affected relatives and concluded that the risk of inheriting the mutation in their family exceeded 50%. Informants reported informing first-degree relatives and regarded it as the parents’ duty to inform children. Parents found it difficult to inform children, however, and had different views on when and how this should be done and expressed a need for updated information and support. In 4 families with few cancer cases, parents tended to inform their children later, because of uncertainty about what information to provide. As an example, a father described planning to talk to his daughter when she turned 18, but at that time felt uncertain of how to bring up the issue and postponed information, whereafter she learnt about Lynch syndrome herself at age 25. Families in which cancer had presented at a young age tended to inform children at a lower age, often referring to family history:

"“/…/dad’s mother actually also had the gene/…/then he [dad] was only 12 years old when she died/…/then dad got it and then his sister got it so it’s more like I’ve pointed out this way for them [the children] and shown that it can be positive to know in order to prevent.” (4/7, mutation carrier, 45 years)"

#### Open or restricted communication

Communication patterns differed within and between the families. Some families reported open communication, e.g. talking to children about heredity, informing new family members, being open about feeling sick and sharing surveillance results. Experiences of cancer were reported to lead to openness and increased awareness:

"“We’ve always been open in the family. We don’t hide anything from each other. Especially since we have experience since my mother has had cancer so many times/…/” (7/16, mutation carrier, 39 years)"

Other families described restricted communication, linked to dysfunctional relationships, as exemplified by a father and mutation carrier who after a divorce lost contact with his son and did not know whether he had undergone genetic testing. Carriers described difficulties in conveying information about genetic testing to relatives with whom they had little contact, which sometimes resulted in information arrest. Their first contact with a distant relative could refer to hereditary cancer, where after they lost contact and did not know whether the relative had undergone genetic testing or if the information had been further spread in the family:

"“I told her [cousin] what had happened [genetic test] and what it was and then she wanted to do the examination/…/Then I don’t know if she did it. I haven’t met her again and then she moved. It isn’t a cousin that I have contact with exactly.” (7/20, mutation carrier, 45 years)"

Other family members referred to shared experiences and chose to attend genetic testing together:

"“/…/it was my sister and I who were involved in this and I felt that I could have the discussion better with her, since we grew up with dad/…/” (4/7, mutation carrier, 45 years)"

Several family members described how they had talked about heredity when they first learned about it but as time went by discussed it only when informing new relatives or children and at the time of surveillance. At-risk individuals who had tested negatively perceived heredity as less relevant and chose not to be involved in disseminating information.

### Influence on family relations and individual roles

Altered relations and roles were discussed by the families with referral to *improved as well as impaired relations*. Furthermore, reference was made to roles of *coordinators* and the importance of *family support*.

#### Improved and impaired relations

Learning about hereditary cancer was considered to influence family relations, though the impact was not necessarily negative. One family with experience of cancer at a young age described how family relations had improved thanks to altered priorities, i.e. not wasting energy on arguments. In contrast, guilt was referred to in relation to passing on a cancer-predisposing mutation as well as among individuals who did not inherit the mutation. Isolation and a negative influence on the marital relationship were described by a spouse:

"“I have seen a big change in her [wife] since the operation and it is not the change that I expected, it is quite the opposite actually/…/we are very hurt with each other and she has become isolated” (1/2, spouse, 51 years)"

#### Coordinators and family support

In several families, one person took on the responsibility for information and reminders about surveillance. In 8 of the 9 families, a female family member took up this role and this individual was typically a mutation carrier or an individual affected by cancer. . Indeed, one family described coordinated reporting of results from genetic testing and surveillance to a female mutation carrier who collected and updated the information. Coordination was by some of the individuals responsible described as demanding, e.g. related to providing support when family members fell ill:

"“Sometimes I also want to be able to be little and scared. But it doesn’t work. It’s…you take it for granted that you’ll have the strength to stand. I’ve had problems with this during periods when relatives have been sick, it easily becomes too much.” (5/10, spouse, 54 years)"

Support from family members was perceived as important in coping with risks posed by heredity. Spouses reported frequently worrying about their affected partner’s cancer risk and looking for signs of illness:

"“…I’ve always been worried about him [husband]…and I think I’ve been thinking more about it [brain tumor] than he has… when he sometimes says “my head is hurting in a strange way” …you know … stuff like that…I guess I’m a little too apprehensive” (6/18, spouse, 34 years)"

Principles for sharing genetic information with individuals outside of the families differed and was reported by 7/27 informants. Professional support was reported to positively influence adherence to surveillance and defined responsibility from one physician was reported to reduce distress.

## Discussions

We assessed family perspectives of hereditary cancer using open-ended interviews with 27 individuals from Lynch syndrome families. Transition to a family at increased risk, patterns of communication, impact on family relations and individual roles were identified as major themes. It should be stressed that there is a variety of outcomes in this kind of study and that counsellors should be aware of the individuality of family member’s responses. Learning about hereditary cancer had implications on several aspects, from worries about cancer to a communication burden. Experiential knowledge was important in this process. Families who had experienced cancer at a young age tended to inform children at a young age and encouraged genetic testing shortly after learning about heredity, which indicates illness and death as major motivators for genetic testing and surveillance [16]. In contrast, individuals in families with few cancer cases or late onset reported finding it difficult to inform children and. young adults in such families expressed weak motivation and tended to postpone genetic testing and participation in surveillance programs. With increasing use of molecular screening and efficient prevention of cancer in families undergoing surveillance, present and future generations in Lynch syndrome families are likely to experience less cancer. Our findings suggest that extended information on the natural history of Lynch syndrome may be required to motivate individuals in such families for genetic testing and participation in surveillance programs [17,18]. Experiences from routine examinations of patients with colorectal and endometrial cancer suggest that, although family history and analysis of tumor tissue suggested Lynch syndrome, only 1 in 5 patients made an appointment with a genetic counsellor [19,20].

Individuals in families with Lynch syndrome are trusted with a large and complex information burden, which can be questioned since dissemination of information occurs through routes and methods determined by family members and is sensitive to personal interpretations and misunderstandings. Communication plays a significant role in managing life at increased risk of cancer and is likely linked to participation in surveillance programs and thereby to the possibilities of reducing morbidity and mortality from colorectal cancer [11,21-25]. Misconceptions were identified among both carriers and non-carriers and were related to children’s risks and the outcome of genetic testing. Since misunderstandings can be resolved by updated information, continued contact with the families related to risk estimates and surveillance may be useful. Open communication has been found to facilitate the spread of information, reduce the risk of misconceptions and ease adjustment to difficult situations [26]. Experience of cancer seems to facilitate openness, which has also been observed in hereditary breast and ovarian cancer [27,28]. In contrast, poor communication is a major determinant of adverse consequences and lack of support from family members can increase vulnerability [26,29].

The influence on family relationships was repeatedly referred to. Positive implications were more often described by carriers than non-carriers, which may reflect adaptation and coping [12,30]. A minority of the families reported a negative impact, including isolation and guilt [26]. Females often took on a coordinating and supportive role and may thus represent a target group for e.g. education regarding communication skills and updated information, which is consistent with observations that females are crucial in communicating genetic information [8,31].

## Conclusions

Genetic counselling emphasizes the individual’s decision to undergo genetic testing and surveillance, but at the same time family relations influence information spread, coping and adherence to surveillance. Perspectives related to becoming a risk family, altered relations and impact on relations was referred to by several family members suggesting that these issues could be relevant to discuss during genetic counselling. The observation that the families are left with a large information burden, which is typically centered around a female member underlines that knowledge about hereditary cancer is sensitive to communication skills and family relations. Our findings suggest a need for updated information and education, which could also counteract the observation that families with fewer cancer cases and later age at onset tend to postpone information, which may have undesired consequences related to the efficacy of surveillance.

## Competing of interest

No part of these data have been sent or published elsewhere. None of the authors have any conflicts of interest.

## Author contributions

KB designed the study, interviewed the participants, analyzed the data and drafted the manuscript. MN designed the study, analyzed the data and drafted the manuscript. CC designed the study, interviewed the participants, analyzed the data and drafted the manuscript. All authors read and approved the final manuscript.
